# Same Day Discharge for Heller Myotomy: Setting Realistic Patient Expectations Is Key

**DOI:** 10.1002/wjs.70047

**Published:** 2025-08-16

**Authors:** Sarah K. Thompson

**Affiliations:** ^1^ College of Medicine and Public Health Flinders University Australia and Adelaide Gastrointestinal Specialists Eastwood South Australia Australia

**Keywords:** achalasia, cardiomyotomy, heller myotomy, surgical outcomes

I read with interest the article by Lopez et al. [1] in this issue entitled: “Same day discharge after minimally invasive Heller myotomy: one surgeon's experience” [1]. The authors performed a retrospective review of 157 consecutive patients who underwent a laparoscopic (*n* = 35) or robotic (*n* = 122) Heller myotomy and Dor fundoplication for type I or II achalasia over a 6‐year period. A surprising 132 patients (84% of the total number) were discharged home on the day of surgery, with only 4 returning to hospital within 48 h of discharge. There were no mortalities and an overall complication rate of 2.6%. The authors concluded that same day discharge following minimally invasive Heller myotomy is safe and feasible, with significant cost and resource benefits.

There is no doubt that Lopez and co‐authors must be commended on their study results. Over the 6‐year period, their same day discharge rate increased from 58% in the first quartile to 92% in the last quartile. This high success rate was in part due to time spent by the surgeon, setting realistic patient expectations following the operation. In particular, a discussion about shoulder tip and neck pain due to CO_2_ insufflation. Another critical aspect of the authors' protocol was a phone call check‐in by the operating surgeon on the first postoperative day. This would have provided much‐needed reassurance to patients and prevented some unnecessary emergency room visits.

When per‐oral endoscopic myotomy (POEM) first emerged as an innovative treatment strategy for achalasia in 2007 [2], one of its main selling points compared to Heller myotomy was a shorter length of stay and reduced hospital costs. For example, in Bayani et al.'s study which compared 64 Heller myotomies to 37 POEM procedures, they found a significantly higher mean hospitalization rate (2.2 vs. 1.1 days, *p* < 0.0001) in the former group [3]. However, the current study effectively debunks this potential advantage. And, looking at more recent systematic reviews, there is no longer a significant difference noted in length of stay between laparoscopic Heller myotomy and POEM [4].

Three other points are worthy of further discussion. First, of the four complications encountered (2.6% of the total cohort), three of these were aspiration events. Although not specified in the article, these aspiration events all occurred on induction. The protocol followed in this study was published in 2022 [5]. The same‐day home recovery protocol described is relevant for patients undergoing one of 3 benign foregut procedures: fundoplication, hiatal hernia repair, and Heller myotomy. However, patients with achalasia are a unique group. Unlike patients with reflux, they have an overly competent lower esophageal sphincter and may have a dilated, tortuous esophagus prone to poor emptying. Therefore, a modification to the protocol may be advisable to avoid aspiration. Namely, fluids only for 24–48 h prior to the day of surgery as well as routine rapid sequence intubation by a skilled anesthesiologist.

Second, the authors' decision to exclude intraoperative endoscopy deserves further comment. In our center, we use intraoperative endoscopy for three reasons: (1) to identify the squamo‐columnar junction to plan extension of the myotomy at least 2 cm onto the stomach, (2) to confirm an adequate myotomy with an open junction, and (3) to exclude a mucosal injury. The authors have used EndoFLIP instead which involves the use of a functional lumen imaging probe (FLIP) containing impedance planimetry electrodes [6]. The FLIP was used to measure the distensibility index (DI) (a ratio of the cross‐sectional area of the lower esophageal sphincter to pressure) during the creation of the myotomy. The authors targeted a DI within a specific target range (> l2.8–5.0 mm^2^/mmHg) to indicate an adequate myotomy [5]. Using this technology, the authors were able to confidently determine the location of the squamocolumnar junction and assess the adequacy of the myotomy. However, the FLIP does not confirm mucosal integrity and it is possible that intraoperative endoscopy would have avoided a late presentation of an esophageal leak, as seen in this patient cohort.

The third point is the lack of postoperative contrast swallows in the current protocol. In our institution, a contrast swallow is obtained on postoperative day one to provide immediate quality assurance that the junction is open and contrast flows freely into the stomach. A contrast swallow can also rule out a leak although it will not identify a thermal injury within 24 h of surgery. There is no doubt that this test is operator‐dependent and it can be resource‐heavy to acquire, especially on the weekend. In a same‐day home recovery protocol, it may be feasible to obtain an outpatient study on day one which could be incorporated into the surgeon's postoperative check‐in.

In closing, Lopez et al. have published a well‐designed study, using a same‐day home recovery protocol for benign foregut procedures. The authors have shown that it is indeed safe and feasible to perform laparoscopic Heller myotomy as a day procedure. The keys to success include careful pre‐planning to set realistic patient expectations prior to surgery and a postoperative day one phone call from the operating surgeon. In a time where hospital beds are at a premium, this study leads the way for other institutions to adopt a same‐day home recovery protocol.

## Author Contributions

**Sarah K. Thompson:** conceptualization, resources, software, writing – original draft, writing – review and editing.

## Conflicts of Interest

The author declares no conflicts of interest.
